# Establishing navigated UBE in Europe – technical note

**DOI:** 10.1016/j.bas.2026.106107

**Published:** 2026-05-26

**Authors:** Nicole Lange, Carolin Albrecht, Raimunde Liang, Luisa Mona Kraus, Ghaith Altawalbeh, Shadi Abulhala, Ann-Kathrin Joerger, Bernhard Meyer

**Affiliations:** Department of Neurosurgery, TUM School of Medicine and Health, Technical University Munich, Germany

**Keywords:** Endoscopic spine surgery, UBE, MIS, Lumbar disc herniation, Intraoperative 3D imaging, Intraoperative navigation

## Abstract

**Introduction:**

Unilateral biportal endoscopic (UBE) lumbar decompression has emerged as a minimally invasive technique for the treatment of degenerative lumbar pathologies, including disc herniation and spinal canal stenosis.

The reproducibility of this technique depends on standardized positioning, imaging, portal placement and stepwise decompression under continuous endoscopic visualization. We present a navigation-assisted workflow that integrates intraoperative three-dimensional imaging (Loop-X, Brainlab AG, Munich, Germany).

**Technique:**

Patients are positioned prone on a radiolucent table. A pelvic reference array is secured prior to skin incision, followed by intraoperative 3D fluoroscopy. Based on navigational planning, two paramedian skin incisions are created to establish a dedicated viewing portal and a separate working portal. Navigation-guided trajectory planning enables controlled access to the interlaminar window and targeted osseous decompression while preserving surrounding anatomical structures. Stepwise decompression of neural elements is performed under continuous irrigation and endoscopic visualization, with intraoperative confirmation of adequate decompression.

**Conclusion:**

Navigation-assisted UBE for lumbar decompression with intraoperative 3D imaging allows precise trajectory planning and controlled portal placement, potentially enhancing reproducibility while maintaining the benefits of minimally invasive surgery.

## Highlights

• Navigation-assisted UBE lumbar decompression is technically feasible.
• A single low-dose intraoperative 3D scan enables precise trajectory planning.
• Real-time navigation allows visualization of endoscope and instruments.
• Implementation involves an initial learning curve and increased setup time compared to conventional techniques.

## 1 Introduction

Minimally invasive spinal surgical (MISS) techniques have become an integral part in the management of degenerative lumbar pathology and are increasingly adopted to reduce access-related tissue trauma and blood loss (Simpson et al., 2022). Additional to minimizing incision length, these approaches aim to preserve paraspinal musculature, reduce soft-tissue disruption and thus decrease postoperative pain and may facilitate faster functional recovery compared to conventional open microsurgical techniques (Chung et al., 2021) (Barber et al., 2019).

Initially described in 1988 (Kambin, 1991) for the endoscopic treatment of lumbar disc herniations, endoscopic spine surgery (ESS) has evolved into an established surgical technique for a wide range of degenerative lumbar pathologies (Hofstetter et al., 2020). Today, ESS is widely regarded as a non-inferior option for the treatment of lumbar disc herniations and spinal stenosis (Chung et al., 2021). More recently, endoscopic techniques have also been explored for spinal instrumentation as well as for the management of tumors and infections (Mayer, 2019) (Telfeian et al., 2015).

Contemporary ESS encompasses both monoportal full-endoscopic techniques and unilateral biportal endoscopy (UBE), which utilizes separate portals for visualization and instrumentation (Simpson et al., 2022) (Hofstetter et al., 2020). Although these approaches have the same goal of minimizing tissue disruption, they differ in terms of surgical handling and workflow (Simpson et al., 2022).

Intraoperative cone-beam CT and navigation can be used for surgical planning and guidance (Oyelese et al., 2018). These tools allow three-dimensional visualization, trajectory planning and accurate level localization (Oyelese et al., 2018) (Assaker et al., 2001). However, data on the integration of CT-based navigation into UBE in lumbar decompression remain limited.

The transition to endoscopic techniques may initially result in longer operating times (Nowitzke, 2005; Nomura and Yoshida, 2017; Hsu et al., 2013). However, several studies have demonstrated that operative time and complication rates improve significantly after an initial series of 20-50 cases (Nomura and Yoshida, 2017) (Qin et al., 2018).

As surgeons become more familiar with the technique, the handling of the endoscope, working channel instrumentation and endoscopic orientation becomes more intuitive and operating times equal or fall below those of conventional microsurgical discectomy (Nomura and Yoshida, 2017) (Zhang et al., 2018) (Xu et al., 2014). Navigation is still not commonly used in combination with UBE, but presents extensive advantages, once it becomes part of routine practice. Therefore, this technical note describes our standardized workflow for navigation-assisted UBE, including patient positioning, imaging acquisition and navigated decompression.

## 2 Methods

### 2.1 Institutional experience

The present technical description is based on a consecutive, single-center series of 36 patients treated with navigated UBE between November 2025 and May 2026. The cohort included 19 female and 17 male patients with a mean age of 50 ± 18 years. Indications included 26 (72%) lumbar disc herniations, 5 facet joint cysts (14%), 4 (11%) central stenoses and 1 (3%) recess stenosis, with 3 cases (8%) involving extraforaminal disc components. Three patients (8%) underwent two-level procedures, while the remaining 33 patients were operated on one level.

### 2.2 Surgical technique

#### 2.2.1 Patient positioning and operating room setup

**Fig. 1:**
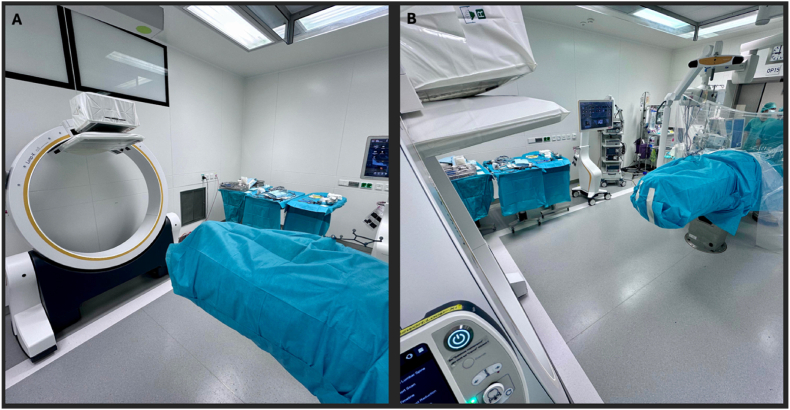
Operating room setup. (A) The Loop-X system (Brainlab neuronavigation, Brainlab AG, Munich, Germany) is positioned at the cranial end of the operating table. (B) The navigation workstation and endoscopic tower are placed contralateral to the surgical approach to allow simultaneous visualization of navigation data and endoscopic imaging.

**Fig. 2:**
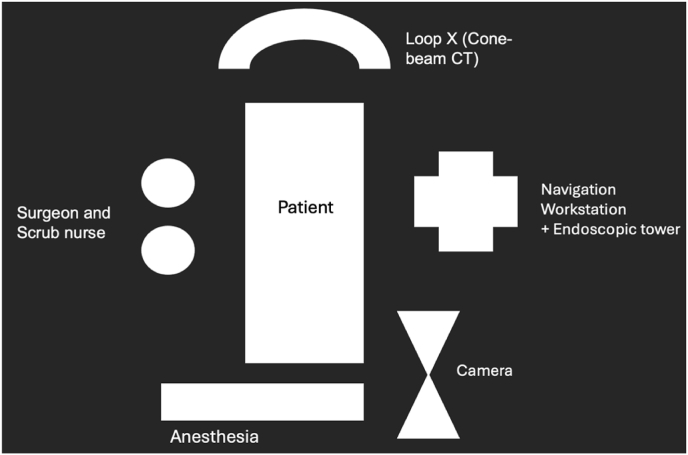
Schematic representation of the operating room.

All procedures are performed under general anesthesia. The patient is initially positioned prone on a radiolucent operating table in a non-sterile setting. Symmetrical thoracic and pelvic support is used for controlled lumbar flexion and widening of the interlaminar window. The abdomen hangs freely and all bony prominences are carefully padded. The arms are positioned alongside the torso and secured. The head is oriented toward the end of the operating room where the Loop-X is positioned (see Fig. 1, Fig. 2).

**Fig. 3:**
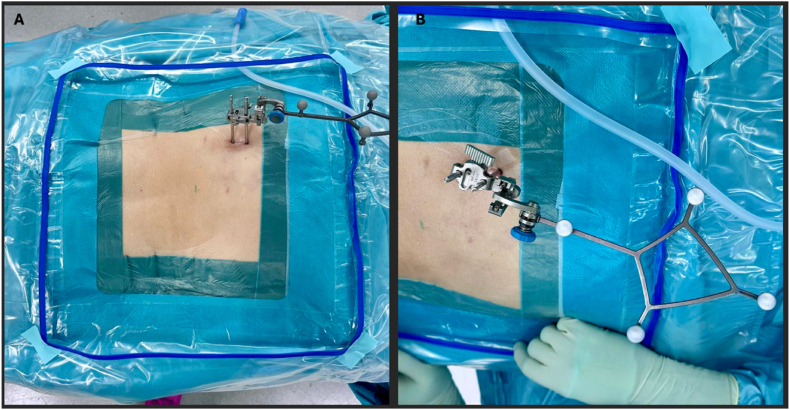
(A) Sterile draping of the operative field. (B) Fixation of the navigation reference array to the right iliac crest.

After final verification of positioning, sterile skin preparation and draping are performed. The pelvic reference array is then fixed under sterile conditions (see Fig. 3).

#### 2.2.2 Intraoperative 3D imaging and navigation

**Fig. 4:**
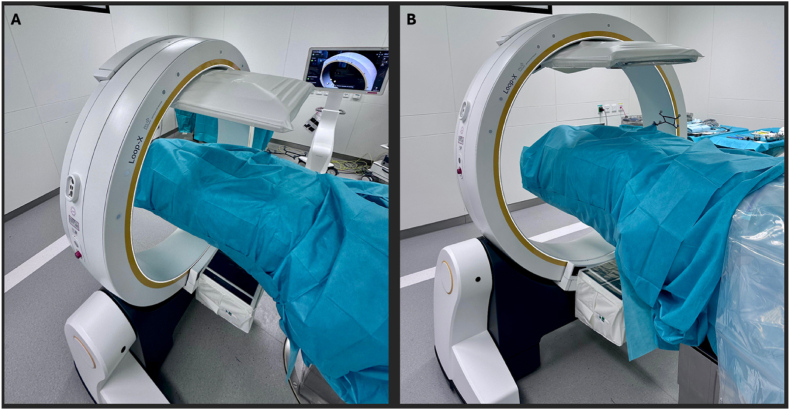
Loop-X positioning and intraoperative 3D acquisition. (A) The ring-shaped Loop-X gantry positioned around the prone patient prior to scanning. (B) Intraoperative cone-beam CT acquisition of the lumbar spine.

Intraoperative three-dimensional imaging is performed using the Loop-X (Brainlab AG, Munich, Germany) in combination with the Brainlab Spine Navigation system (see Fig. 4). To reduce radiation exposure, the CT acquisition volume is limited to the required levels only.

#### 2.2.3 Surgical steps

1) Skin incision and access

**Fig. 5:**
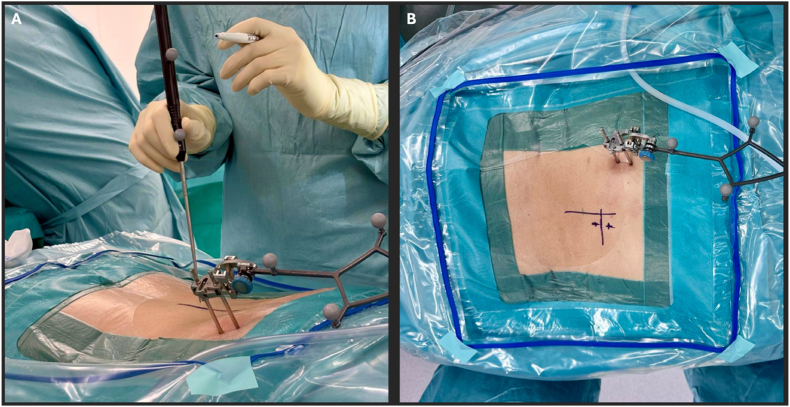
(A) Navigation pointer used to confirm the planned entry point and working trajectory. (B) Marking of the final skin incisions according to the navigated trajectory.

After confirmation of the target segment and planned trajectory using navigation, two paramedian skin incisions of approximately 10 mm are created to establish a dedicated viewing portal and a separate working portal (see Fig. 5).

**Fig. 6:**
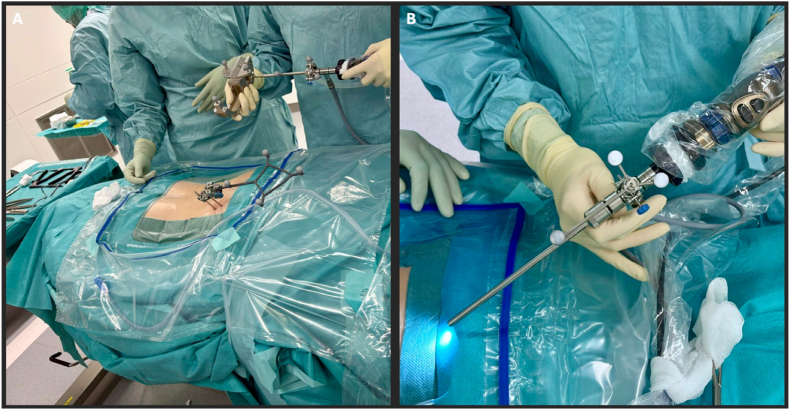
(A) Calibration of the endoscopic working trocar within the navigation system prior to insertion. (B) Endoscopic trocar with the camera used for visualization.

The reference array is attached to the endoscope and calibrated (see Fig. 6). Under navigational guidance, the interlaminar window is precisely localized and portal positioning is adjusted to allow optimal access to the planned segment.

2) Intraoperative guidance

**Fig. 7:**
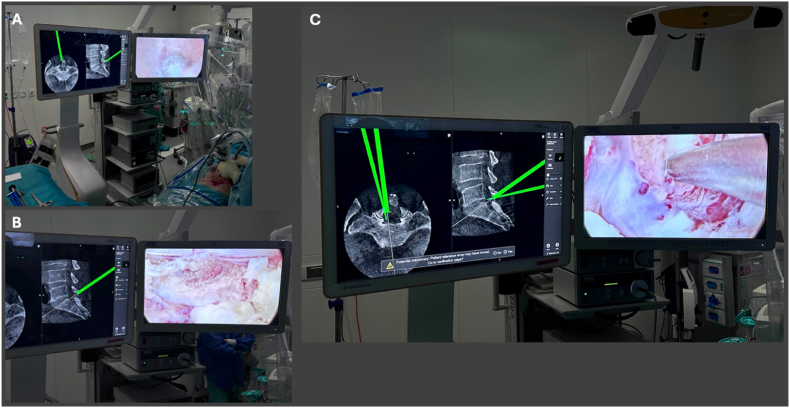
Stepwise endoscopic decompression under navigation guidance with axial and sagittal reconstructions obtained with the Loop-X system**.** (A–B) Navigation-guided drilling with continuous real-time visualization of the endoscope tip and viewing axis within the CT dataset. (C) Anatomical verification using a navigated pointer for spatial correlation within the intraoperative imaging.

**Fig. 8:**
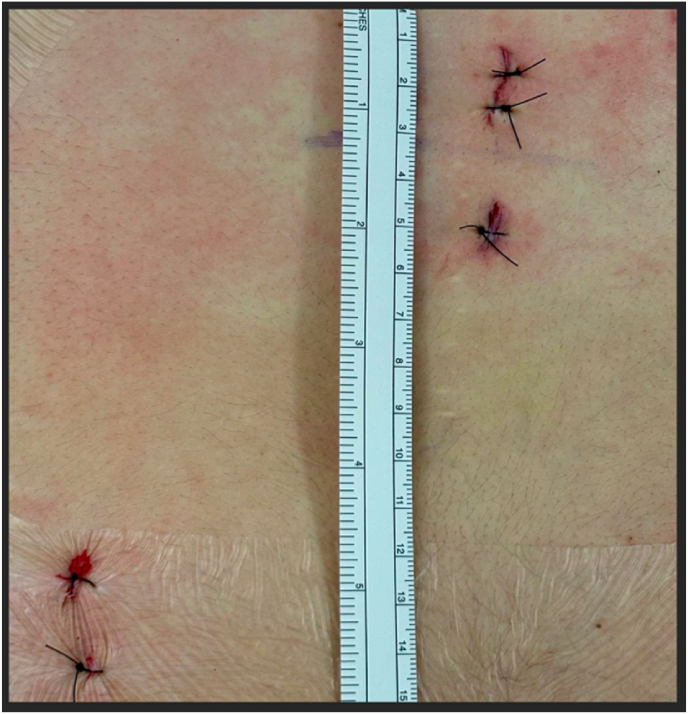
Skin closure: The two stab incisions for navigation reference fixation (left) and the two working channel incisions (right) are closed with single interrupted sutures.

The calibrated endoscope is continuously tracked within the navigation system. Thereby, any further imaging is obsolete and wearing leads is not necessary. The tip position and optical axis are visualized in real time within the intraoperative imaging dataset (see Fig. 7), allowing direct correlation between the endoscopic field of view and the corresponding anatomical structures. When required, a navigated pointer can be used to verify the anatomical location within the intraoperative dataset. Both instruments are simultaneously visualized within the navigation interface providing precise spatial orientation during decompression. Intraoperative screenshots enable digital documentation of the whole process, as for example control of adequate bony decompression (see Fig. 8).

3) Hemostasis and Closure

After final inspection confirming adequate neural decompression and absence of active bleeding, the endoscope and working instruments are withdrawn sequentially under direct visualization. The pelvic reference array is subsequently removed. Both portal incisions and both stab incisions from the array are closed with interrupted sutures, followed by application of a sterile compressive dressing.

#### 2.2.4 Postoperative management

Patients are usually mobilized on the day of the surgery. Postoperative analgesia and thromboprophylaxis are administered in accordance with institutional protocols. Hospital discharge is typically performed on postoperative day 1, provided adequate pain control, independent mobilization and stable neurological findings are confirmed. Skin sutures are removed after 7 days.

### 2.3 Technical advantages

#### 2.3.1 Reduction in radiation dose

A single low-dose intraoperative 3D acquisition was obtained in all cases prior to decompression. No additional scans were required. Continuous fluoroscopy was not used at any stage of the procedure. During image acquisition, the operating team left the radiation field, and no intraoperative radiation exposure occurred to the surgical staff. Effective dose was lower than 1.16 mSv per scan.

#### 2.3.2 Real-time visualization

Continuous tracking and real-time visualization of the endoscope tip and viewing axis within the intraoperative 3D dataset were available throughout decompression in all cases. Simultaneous display of both instruments within the navigation interface was feasible in all procedures.

#### 2.3.3 Digital documentation

Intraoperative navigation screenshots enabled digital documentation when required.

#### 2.3.4 Extent of bone resection

In comparison to the standard microscopic interlaminar approach at our institution, the intraoperative bony window was reduced in extent, preserving midline structures and facet joints, as targeted resection is done.

## 3 Preliminary clinical results

In our series of 36 patients, the median operative time was 101.5 min (range 47-306 min) and blood loss was negligible in all cases. Immediate postoperative pain relief was achieved in 94% (n = 34) of patients. Of eight patients presenting with preoperative motor deficits, 75% (n = 6) showed postoperative improvement, while deficits persisted in two patients (25%), both of whom also reported no improvement in radicular pain.

One of the patients subsequently underwent open microsurgical revision, while the other was indicated for reoperation but opted for continued conservative management.

Regarding complications, two patients (5.6%) required revision surgery: one due to a persistent cerebrospinal fluid (CSF) leak and one due to early recurrent disc herniation. Notably, the patient requiring revision for CSF leak also represented the only case requiring intraoperative conversion to open microsurgical decompression. One additional CSF leak (2.8%) was managed conservatively. No wound healing disturbances were observed, and patients were typically discharged on the first postoperative day.

## 4 Discussion

Navigation-assisted unilateral biportal endoscopic lumbar surgery is feasible and technically reproducible. The endoscopic technique minimizes soft tissue trauma due to the small working channels resulting in limited paraspinal muscle injury and negligible blood loss (Hofstetter et al., 2020). Compared with conventional microsurgical sequestrectomy, the skin incisions and tissue corridor are smaller and continuous endoscopic visualization allows for targeted decompression under direct vision.

The integration of intraoperative 3D imaging with navigation enhances spatial orientation (Assaker et al., 2001). This is particularly important in anatomically demanding cases, e.g., high iliac crest at L5/S1, narrow interlaminar window, or obese patients.

The navigated trajectory planning may facilitate accurate working channel placement and potentially reduce repeated fluoroscopic adjustments (Assaker et al., 2001).

Furthermore, during the initial implementation of the workflow, operative times may exceed those of established microsurgical techniques (Nomura and Yoshida, 2017). In the early adoption phase of navigation-assisted UBE, additional time is required for system setup, reference array placement, intraoperative 3D image acquisition, trajectory planning and familiarization with the technical workflow. However, a reduction in operative time was observed with increasing procedural experience. After a limited number of cases and growing familiarity with the instrumentation and navigation workflow, operative times progressively approached those of conventional microsurgical surgery (Nowitzke, 2005) (Nomura and Yoshida, 2017) (Xu et al., 2014). As with other endoscopic spine techniques, structured workflow standardization and team experience are key determinants of operative efficiency.

When using intraoperative three-dimensional imaging systems such as cone-beam CT-based navigation platforms, radiation exposure must be carefully considered.

In our workflow, a single low-dose Loop-X acquisition is obtained after reference array placement and prior to skin incision. This showed a mean effective dose of 1.16 mSv per scan. Accordingly, published dosimetric analyses of intraoperative cone-beam CT systems in lumbar spine surgery report CTDIvol values typically ranging between approximately 4-8 mGy, corresponding to effective doses of roughly 0.5-1.5 mSv per acquisition (Zhang et al., 2009) (Mendelsohn et al., 2016).

In conventional fluoroscopy-guided procedures at our institution, a minimum of four radiographic control images was typically required for level confirmation and working channel positioning. A single lumbar fluoroscopic image is reported to result in an effective dose of approximately 0.5-1.5 mSv, depending on projection and patient factors. Consequently, four standard control images may correspond to a cumulative dose of about 2 mSv, with higher exposure if repeated checks or continuous fluoroscopy are required. Therefore, by restricting imaging to a single acquisition and avoiding repetitive fluoroscopic confirmation, radiation exposure can be entirely avoided for the surgical staff and even be reduced for the patients.

Beyond that, the navigation-assisted approach offers advantages in surgical orientation in more complex cases. In routine cases with straightforward anatomy, standard fluoroscopy-guided UBE is very efficient. However, it is limited by intermittent two-dimensional visualization. The continuous 3D spatial orientation provided by navigation is especially beneficial for complex anatomical conditions. Variants as Bertolotti decompressions, obese or scoliotic patients, revision cases, multilevel pathologies or even tumor cases can be approached with enhanced precision, as the real-time tracking of the endoscope tip allows for a better identification of landmarks even in altered anatomy. This transition from intermittent checks to continuous guidance potentially increases the reproducibility of the decompression.

Consistent use is essential when implementing navigation-assisted endoscopic decompression. Routine application enables the surgical team to establish a standardized workflow, gain technical familiarity with reference array handling and instrument calibration and develop confidence in interpretation. By integrating navigation as a routine component early on, technical handling and interpretation become internalized before encountering anatomically challenging, more complex cases or revision cases. This ensures that navigation is already well established instead of being introduced during high-demand situations. In addition, this approach may broaden the operative spectrum, facilitating the application of endoscopic techniques to more complex pathologies, such as tumor cases, where enhanced three-dimensional orientation can be particularly valuable.

This technical note demonstrates the feasibility and practical implementation of navigation-assisted UBE in a consecutive single-center series of patients who underwent lumbar decompression. The workflow proved technically reproducible and can be integrated into established endoscopic spine programs. However, conventional microsurgical discectomy remains a highly effective and widely validated standard treatment. Navigation-assisted endoscopic surgery should be considered as a standard technique that may offer workflow advantages, as well as reduced tissue trauma, lower infection rates, and a potential for establishing outpatient surgeries (Simpson et al., 2022) (Chung et al., 2021) (Sharma et al., 2025).

Further prospective comparative studies are required to evaluate potential differences in clinical outcome, complication rates and cost-effectiveness.

### 4.1 Limitations

This report reflects a single-center experience with a limited sample size (n = 36) and therefore primarily addresses technical feasibility rather than clinical efficacy. No comparison with other minimally invasive techniques, uniportal endoscopy or open lumbar decompression was performed and the absence of a control group prevents conclusions being drawn regarding superiority, complication rates or operative times. In addition, systematic long-term follow-up data, including recurrence, reoperation rates and patient-reported outcome measures were not analyzed.

A potential limitation of the presented workflow is that it requires additional infrastructure for navigation and intraoperative 3D imaging. This may hinder its adoption in settings with limited resources. However, the described setup does not rely on proprietary endoscopic systems, as the navigation reference array can be integrated with standard arthroscopic equipment commonly available in many institutions.

Additionally, integrating navigation-based workflows may initially increase operative time, particularly due to setup and imaging. With increasing experience and workflow standardization, this additional time is likely to decrease and become negligible.

## 5 Conclusion

Unilateral biportal endoscopic interlaminar lumbar decompression, assisted by navigation, can be incorporated into a standardized surgical workflow, using three-dimensional, intraoperative imaging with the Loop-X. A single low-dose scan enables accurate trajectory planning while preserving the minimally invasive nature of the procedure. It has been implemented as a standard procedure in our institution.

## Availability of data and materials

Source data are stored at the Department of Neurosurgery, Technical University of Munich, Germany. Requests for raw data or collaboration should be addressed to the corresponding author.

## Author contributions

LN: Conception and design, Data acquisition, Analysis, Drafting the article, Final approval.

CA: Conception and design, Data acquisition, Analysis, Drafting the article, Final approval.

RL: Conception and design, Data acquisition, Analysis, Final approval.

LMK: Conception and design, Revising the article for important intellectual content, Final approval.

GA: Conception and design, Revising the article for important intellectual content, Final approval.

SA: Conception and design, Analysis, Final approval.

AKJ: Conception and design, Revising the article for important intellectual content, Analysis, Final approval.

BM: Conception and design, Revising the article for important intellectual content, Final approval.

## Declaration of generative AI and AI-assisted technologies in the manuscript preparation process

During the preparation of this paper, the authors utilized ChatGPT5.2 (OpenAI, San Francisco, California, USA) to assist with spelling, grammar, and style corrections. Following the use of this tool, the authors thoroughly reviewed and made additional corrections to ensure accuracy and appropriateness. The authors take full responsibility for the final content and conclusions presented in this publication.

## Funding

No funding for research, writing, and/or publication of this article was received by the author(s).

## Declaration of competing interest

The authors declare the following financial interests/personal relationships which may be considered as potential competing interests: Bernhard Meyer reports a relationship with Brainlab SE that includes: consulting or advisory, paid expert testimony, and speaking and lecture fees. Nicole Lange reports a relationship with Nexon Medical that includes: consulting or advisory. Luisa Mona Kraus reports a relationship with Brainlab SE that includes: consulting or advisory. If there are other authors, they declare that they have no known competing financial interests or personal relationships that could have appeared to influence the work reported in this paper.
